# The pigeon circovirus evolution, epidemiology and interaction with the host immune system under One Loft Race rearing conditions

**DOI:** 10.1038/s41598-024-64587-3

**Published:** 2024-06-15

**Authors:** Tomasz Stenzel, Daria Dziewulska, Ewa Łukaszuk, Joy M. Custer, Matthew D. De Koch, Simona Kraberger, Arvind Varsani

**Affiliations:** 1 Department of Poultry Diseases, Faculty of Veterinary Medicine, University of Warmia and Mazury in Olsztyn, Olsztyn, Poland; 2 Biodesign Center for Fundamental and Applied Microbiomics, Center for Evolution and Medicine, School of Life Sciences, Arizona State University, Tempe, USA; 3 Structural Biology Research Unit, Department of Integrative Biomedical Sciences, University of Cape Town, Observatory, Cape Town, South Africa

**Keywords:** ddPCR, Gene expression, One Loft Race, Pigeon circovirus, Recombination, Viral evolution, Viral evolution, Pathogens

## Abstract

This study was aimed to investigate the frequency of PiCV recombination, the kinetics of PiCV viremia and shedding and the correlation between viral replication and host immune response in young pigeons subclinically infected with various PiCV variants and kept under conditions mimicking the OLR system. Fifteen racing pigeons originating from five breeding facilities were housed together for six weeks. Blood and cloacal swab samples were collected from birds every seven days to recover complete PiCV genomes and determine PiCV genetic diversity and recombination dynamics, as well as to assess virus shedding rate, level of viremia, expression of selected genes and level of anti-PiCV antibodies. Three hundred and eighty-eight complete PiCV genomes were obtained and thirteen genotypes were distinguished. Twenty-five recombination events were detected. Recombinants emerged during the first three weeks of the experiment which was consistent with the peak level of viremia and viral shedding. A further decrease in viremia and shedding partially corresponded with IFN-γ and MX1 gene expression and antibody dynamics. Considering the role of OLR pigeon rearing system in spreading infectious agents and allowing their recombination, it would be reasonable to reflect on the relevance of pigeon racing from both an animal welfare and epidemiological perspective.

## Introduction

Racing pigeons are popular domestic animals which are kept in numerous countries around the world. The growing popularity of pigeon racing puts a demand on veterinary medicine specialists to expand their knowledge regarding pigeon health. For this reason, the International Veterinary Pigeons Association was founded, bringing together veterinarians from all over the world who focus on pathology of pigeons^1^. These birds require proper feeding, training, husbandry and veterinary care to achieve the best possible results in races. In practical terms, breeding of racing pigeons is a combination of mass breeding, as birds are kept in flocks, with breeding of companion animals, because the market value of an individual bird can be very high, depending on its pedigree, phenotype and racing results^2,3^. This combination is an issue in pigeon breeding, because practices such as keeping birds in multi-age flocks and regular (usually weekly) collective transport of even thousands of birds originating from different breeding facilities for racing create perfect conditions for the spread of infectious agents. The sport known as pigeon racing is based on the controlled release of birds which return to their home loft over a measured distance that depends on the category of the race, e.g. the distance of a sprint race is about 100 km, whereas during the marathon races pigeons have to find the way to their home lofts from a distance of 800–1000 km. The winner is the pigeon that covered the specified distance the fastest—and due to the different destinations of the race, it is not necessarily the first pigeon. In this situation, numerous additional factors affecting the final result, such as good geographical location of certain lofts, different feeding, training and health programs as well as the use of performance-enhancing drugs can occur^4,5^. This was the reason for developing the One Loft Race (OLR) system, which is intended to eliminate the aforementioned factors by keeping young birds from various (in practice, even from several hundred) maternal lofts in one facility so that their training is together and the racing flights have the same destination. This allows for the assumption that the race is truly won by the best pigeon.

However, such a system poses an epidemiological threat for the animals, because keeping as many as a few thousands young birds originating from different breeding facilities together creates the perfect conditions for inter-individual transmission and spreading of various pathogens. The incidence of disease and mortality in OLRs can be very high, because pigeons as young as 2–4 months of age do not yet have a fully developed immune system and are more prone to infections^6^. Moreover, a high rate of inter-individual infections with various pathogens also creates a convenient opportunity for recombination of viruses, especially those which are more prevalent and can cause subclinical infections. A well-known example of such a virus is the pigeon circovirus (PiCV), which is disseminated all around the globe, and its spread is possibly facilitated by pigeon racing as well as the international pigeon trade^7^. Asymptomatic infections with this virus are quite common, and they have been noted in birds of all ages^8–13^. Moreover, a serological study of domestic pigeons from selected breeding flocks from Poland revealed the presence of anti-PiCV antibodies in an average of 72% of birds, regardless of their infection status^12^.

Circoviruses (family *Circoviridae*) have some of the smallest genomes among viruses, which makes them a very valuable material for molecular and bioinformatic research. The PiCV genome consists of single-stranded circular DNA of approximately 2000 nucleotides. PiCVs have an ambisense genome organization containing open reading frames (ORFs) on different strands of replicative ssDNA. The most important ORFs are considered ORF C1, which encodes the capsid protein, and ORF V1, which encodes the replication-associated protein^14–16^. A characteristic feature of PiCV genomes is the high genetic diversity that results from the high rates of substitution and recombination^11,17–20^. Recombination occurs when at least two viral genomes co-infect the same host cell and exchange genetic material. Therefore, recombination does not create new mutations at the nucleotide level but introduces new combinations of the existing ones. As a result, several mutations previously incorporated into a genomic region can be simultaneously transferred to another region by a single recombination event. Recombination can have a major impact on virus evolution, as it has been associated with the expansion of viral host range, the emergence of new viruses, the alteration of transmission vector specificity, the increase in virulence and pathogenicity, the modification of tissue tropism and the evasion of host immunity^21^.

In view of the above, we performed a study to identify the frequency of inter-individual recombination leading to the development of novel PiCV variants and to assess the time necessary for it. We also made every effort to investigate the kinetics of PiCV viremia and shedding, as well as the potential correlation between viral replication and the response of the host immune system in young pigeons naturally infected with PiCV and kept under conditions mimicking the OLR system.

## Results

### Course of the experiment

**Figure 1:**
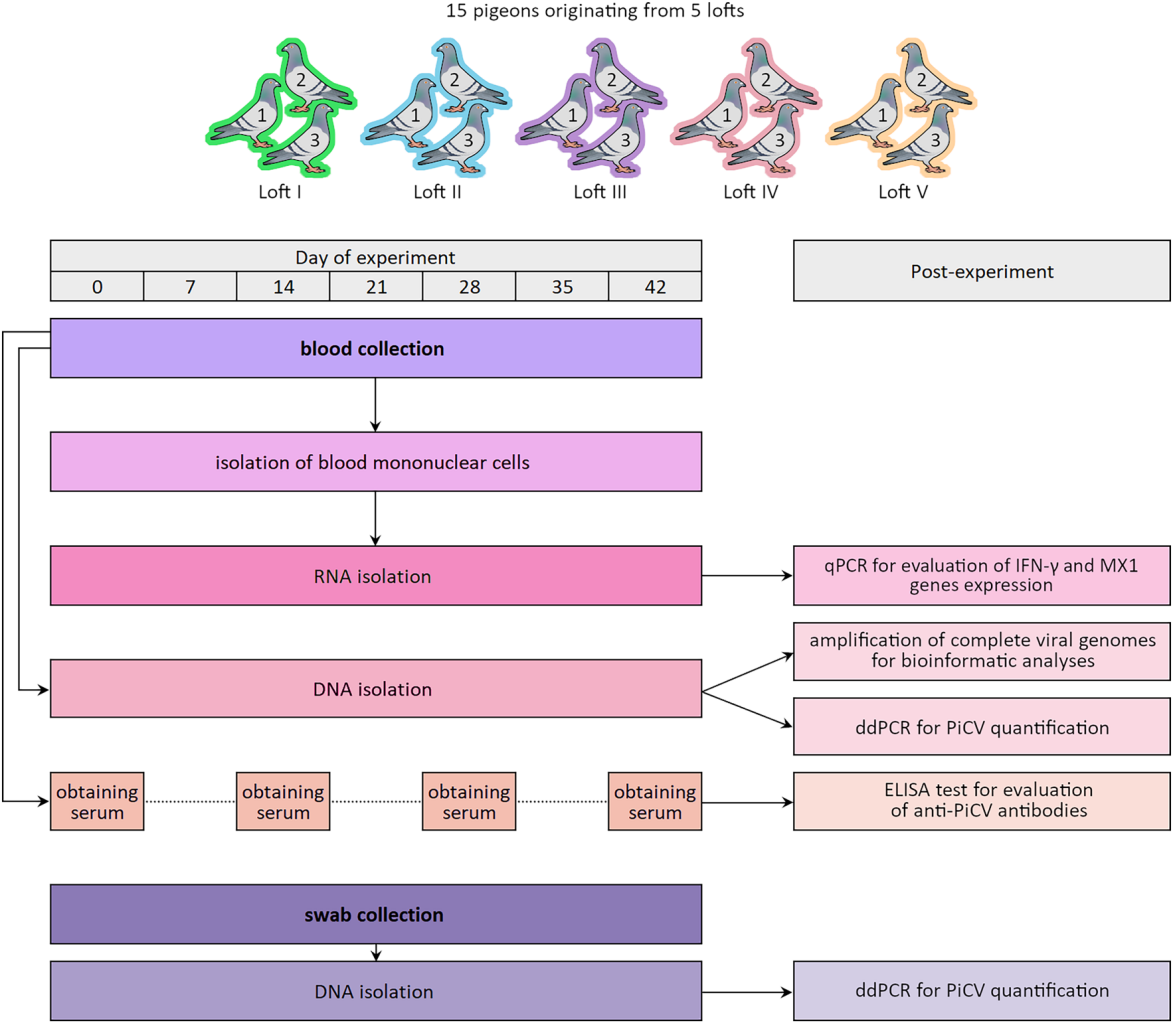
Overview of the experimental schedule.

During the experiment, pigeons did not develop clinical symptoms except for birds number II_2 and V_3, originating from lofts number II and V, respectively. These birds had been showing non-specific clinical symptoms since day 10 of the experiment and died before the third sampling date (pigeon number V_3) and the sixth sampling date (pigeon number II_2). The necropsy of those pigeons revealed cachexia, fibrinous inflammation of air sacs, fibrinous pericarditis and perihepatitis; whereas microbiological examination showed presence of non-hemolytic *E. coli* in organ samples of both birds. The PiCV genome copy number in the blood and cloacal swabs of those pigeons was very high in comparison to the rest of the experimental birds (Supplementary Table S1; Fig. 1).

### PiCV viremia and shedding

**Figure 2:**
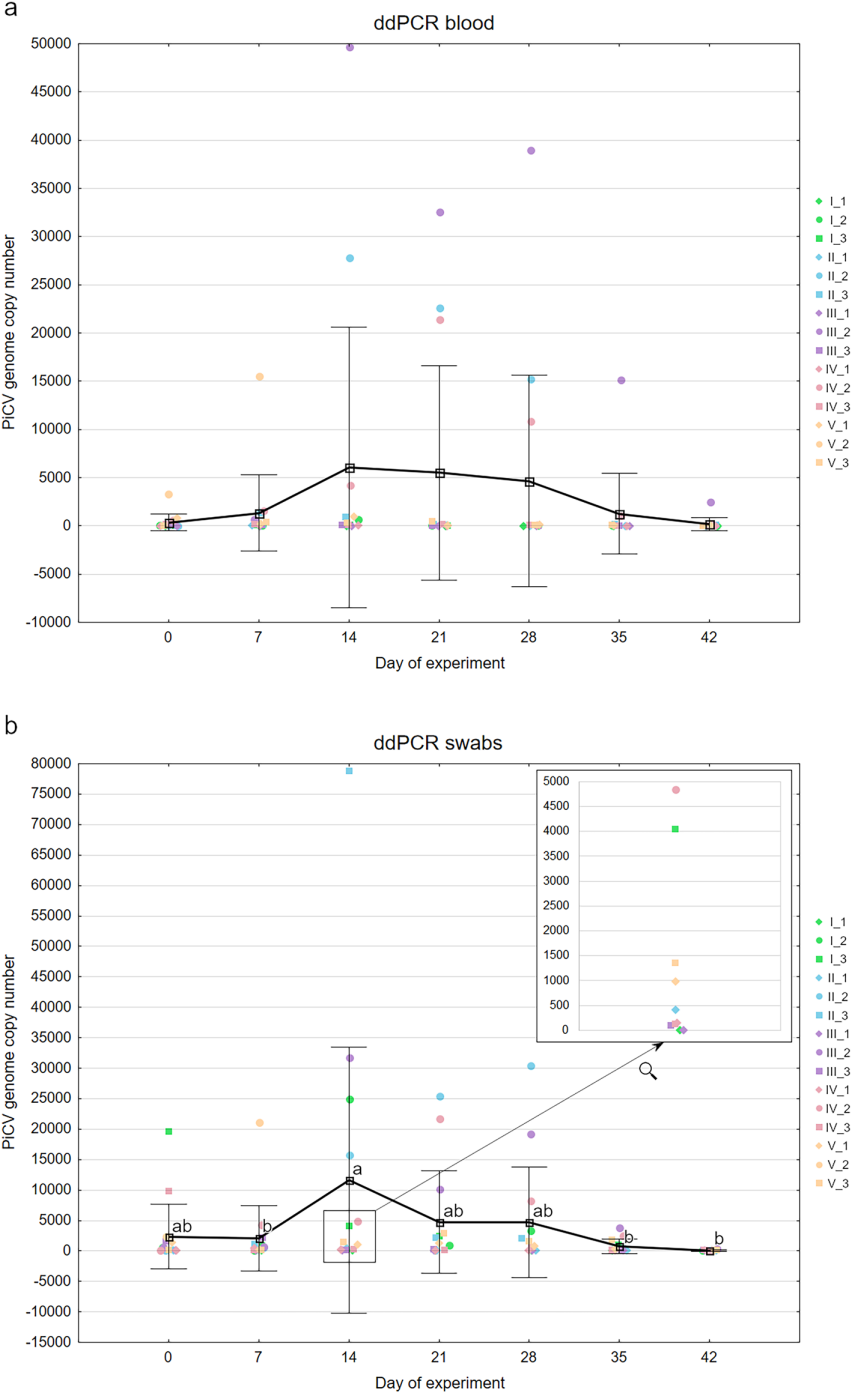
The results of ddPCR pigeon circovirus quantification during the whole experimental period in blood (**a**) and cloacal swab (**b**) samples acquired from the experimental pigeons. The different letters (a,b) indicate a statistically significant difference in PiCV genome copy numbers between the sampling dates (p < 0.05). Whiskers represent the standard deviation values. The results for the individual pigeons are encoded by color according to Fig. 1.

The average PiCV genome copy number in the blood of examined pigeons measured 342 ± 859 at the beginning of the experiment and increased gradually until day 14 of the experiment, reaching 6054 ± 14,556. The viremia oscillated between 4600 and 5500 ± 12,000 for the next two weeks and decreased remarkably starting from day 35 of the experiment, reaching the number of 91 ± 214 on the date of the last sampling (Fig. 2a). Due to the high values of standard deviation, the differences between sampling dates were found statistically insignificant (p = 0.57–1.00).

The average PiCV genome copy number in the cloacal swabs (virus shedding) of the experimental pigeons revealed 2360 ± 5533 at the beginning of the experiment and was the highest on day 14 of the experiment, reaching 11,639 ± 21,851. Following day 14, the PiCV shedding decreased to ca. 4700 ± 8500 for next 2 weeks. The shedding noted on the day of the final sampling was 116 ± 231 PiCV genome copies. The pigeon circovirus shedding on day 14 of the experiment was significantly higher than on day 7 (p = 0.05) and on days 35, 42 and 49 (p = 0.03–0.05) of the experiment (Fig. 2b).

The PiCV genome copy number in the last blood and cloacal swab samplings before death was 22,950 and 83,200 for bird number II_2, and 43,700 and 137,750 for bird number V_2, respectively. In turn, the average PiCV genome copy number in the blood and swab samples for all experimental pigeons was 4665 ± 10,974 and 4706 ± 9025, respectively, on the day the bird number II_2 died; and 1334 ± 3964 and 20,753 ± 5350, respectively, on the day the bird number V_2 died.

### qPCR evaluation for IFN-γ and MX1 genes expression

**Figure 3:**
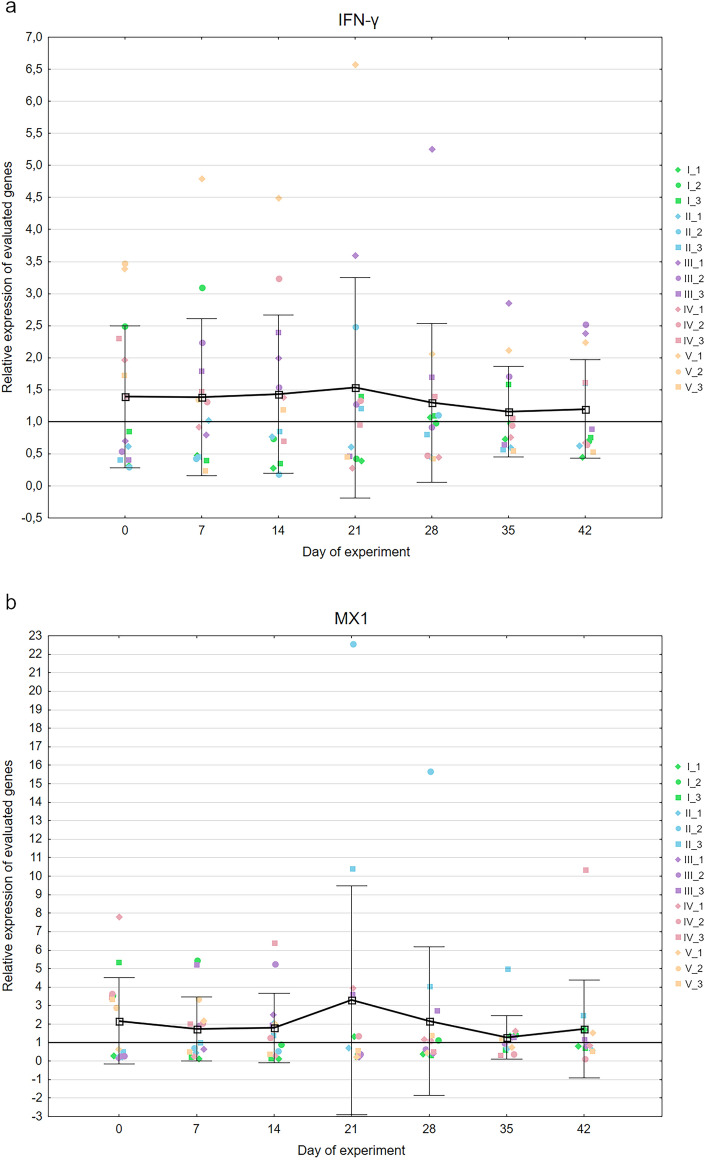

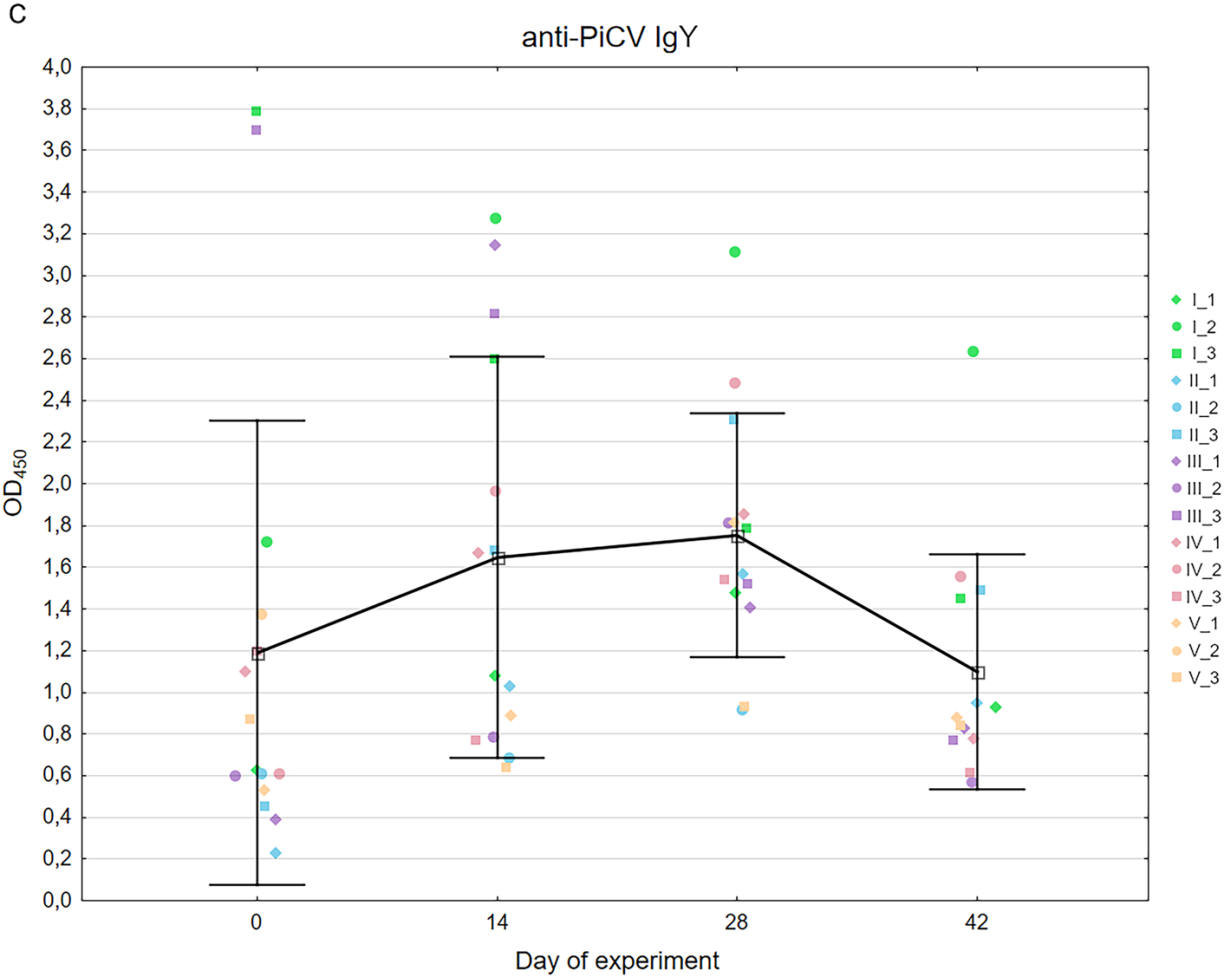
Mean relative expression of the genes encoding IFN-γ (**a**), and MX1 (**b**) as well as in-house ELISA detection of anti-PiCV IgY in sera of the experimental pigeons (**c**) throughout the experiment. The mean relative expression values above 1 (horizontal black line) indicate a higher gene expression compared to the control pigeons. Whiskers represent the standard deviation values. The results for individual pigeons are encoded by color according to Fig. 1.

The relative expression of IFN-γ in the experimental birds was almost constant during the whole experiment and measured 1.16 ± 0.7 to 1.53 ± 1.72. The highest expression was noted on day 21 of the experiment. Similar pattern of expression was noted in case of MX1 gene, which oscillated from 1.29 ± 1.17 to 3.29 ± 6.18 with the expression peak on day 21 of the experiment. (Fig. 3a,b). The differences between expression level in certain sampling dates were statistically insignificant (p = 0.97 and p = 0.99 for MX1 and IFN-γ genes, respectively).

### Kinetics of anti-PiCV IgG in the sera of investigated pigeons

All pigeons were positive for anti-PiCV IgG throughout the whole experiment; however, the mean OD_450_ value was low at the beginning of the experiment and measured 1.21 ± 1.11. The average value of this parameter increased gradually to day 28 of the experiment, reaching 1.82 ± 0.58. On the last day of serum sampling, the OD_450_ value decreased to 1.1 ± 0.56. The difference between values obtained in certain sampling dates was found statistically insignificant (p = 0.14–0.97) (Fig. 3c).

### Bioinformatic analyses

#### Amplification of complete PiCV genomes and genome recovery

The complete PiCV genomes were obtained from 37 blood and 34 cloacal swab samples from different sampling dates, thus recovering 336 and 93 complete PiCV genomes, respectively (Supplementary Table S1). Forty-one identical sequences were removed from further analyses, so all bioinformatic analyses were performed on 388 complete PiCV genome sequences. These sequences were deposited in the GenBank database under the Accession Numbers OR801794-OR802128 and OR999199-OR999278 for blood and swabs, respectively.

#### Diversity of PiCV strains obtained in this study

**Figure 4:**
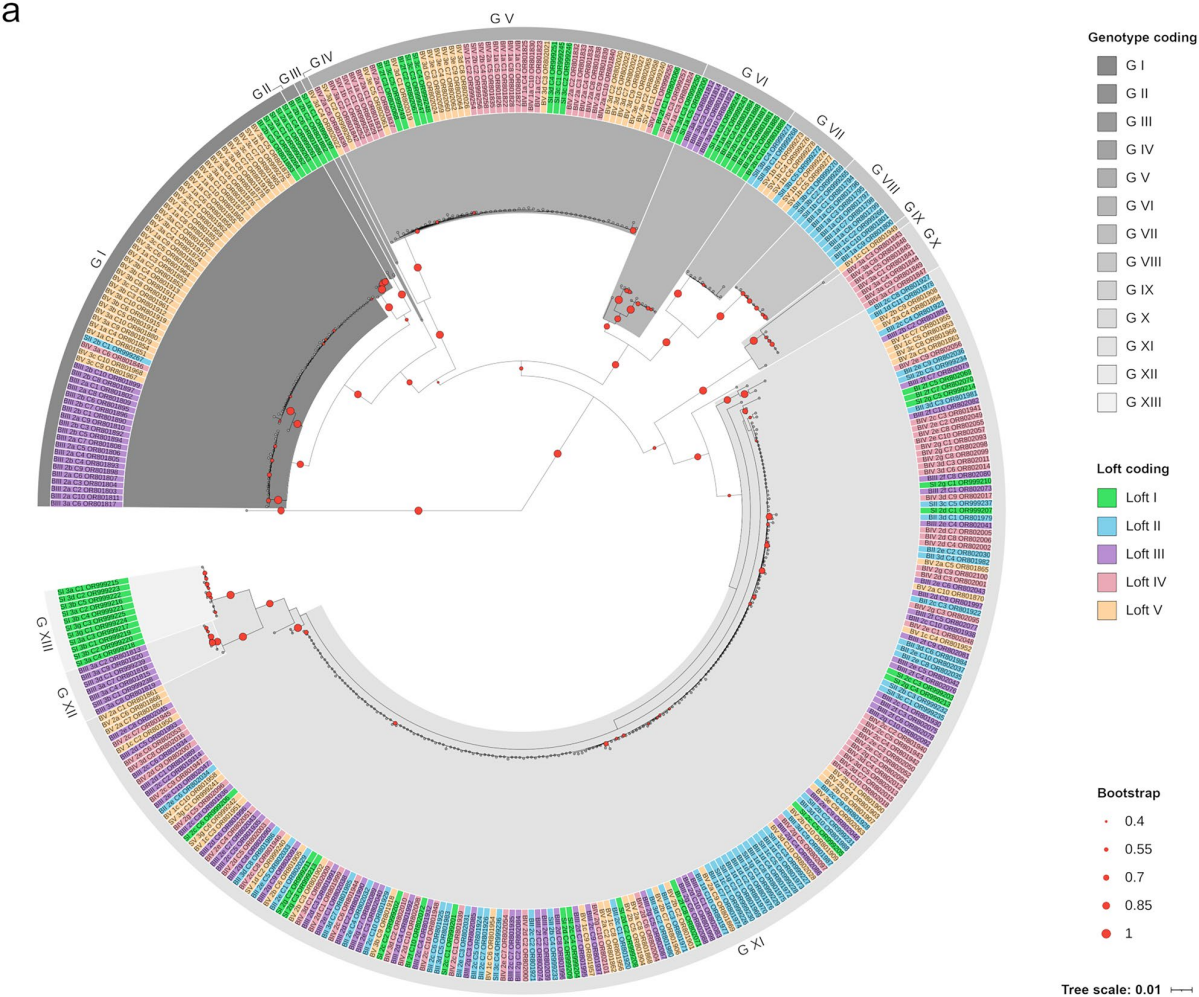

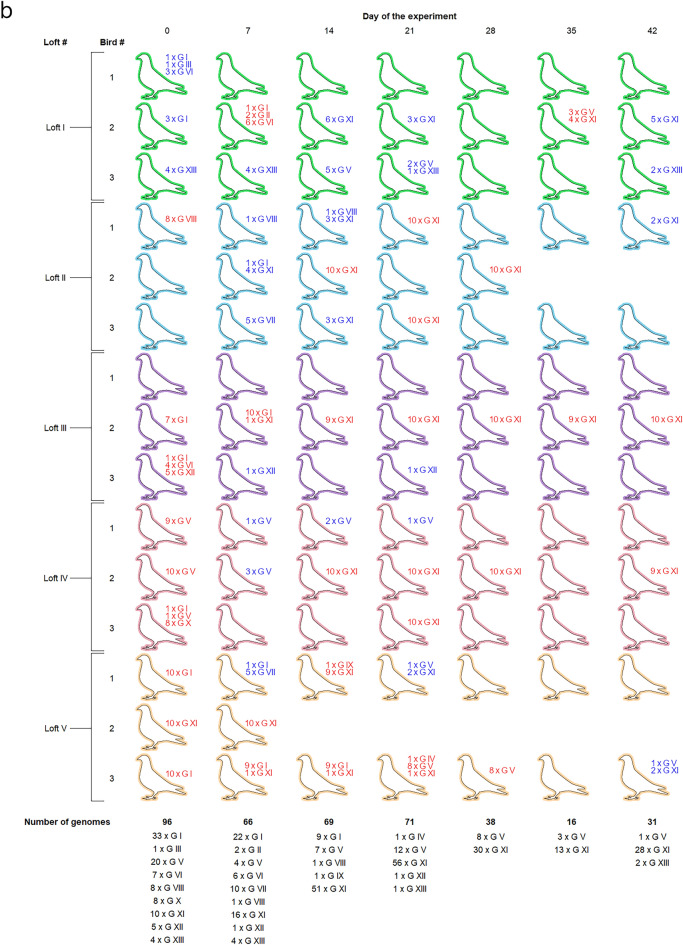
(**a**) The Neighbor-joining tree showing the genetic connections of 388 complete PiCV genome sequences investigated in this study. The sequences were encoded with the following formula: B (blood)/S (swab), loft number (I–V), _pigeon number (1–3), sampling date (a–h), _clone number (C1–10) and the Accession Number. The color marking of individual sequences indicates the loft of origin: green—loft I, blue—loft II, purple—loft III, pink—loft IV and orange—loft V. Sequences belonging to different genotypes are highlighted with different shades of grey. (**b**) PiCV genotypes recognized in the investigated pigeons during the experiment. Lack of symbols near the pigeon shape indicates an unsuccessful recovery of the complete PiCV genome on the specific sampling date. Digits near the selected shapes indicate the number of PiCV clones belonging to the specific genotype (G I–G XIII) detected in blood samples (red digits) and swab samples (blue digits) taken from the experimental birds. The pigeon lofts are encoded by color according to Fig. 1.

The recovered complete PiCV genome sequences showed 92.32% genome-wide pairwise identity (84.41–99.99%). Based on the established threshold for genotype separation, the obtained genome sequences were classified as 13 genotypes (I–XIII) (Fig. 4a).

The highest number of complete PiCV genomes originating from both blood and cloacal swab samples was recovered between the day 0 and 21 of the experiment (96, 66, 69 and 71 genomes on days 0, 7, 14 and 21, respectively). On these sampling dates, the complete PiCV genomes were recovered from at least one pigeon from each of the maternal lofts. The number of genotypes that we were able to recover decreased to 38, 16 and 31 on the day 28, 35 and 42 of the experiment, respectively. Moreover, we were unable to recover complete PiCV genomes from the loft I on the day 28 of the experiment and from the lofts IV and V on the day 35 of the experiment.

At the beginning of the experiment, nine genotypes were present, with genotypes I, V, VII and XI being the most prevalent. Pigeons originating from loft I harbored PiCV belonging to genotypes I, III, VI and XIII. Circoviruses from the genotype VIII were only found in one pigeon from loft II. Birds from loft III harbored PiCV belonging to genotypes I, VI and XII. Pigeons originating from loft IV were carriers of circoviruses belonging to genotype I, V and X, whereas representatives of loft V harbored PiCV from genotypes I and XI. During the whole experimental period, the complete PiCV genome sequences identified as genotypes II, III, IV, IX and X were detected only in single birds during one sampling. Interestingly, starting from the second sampling date, genotype XI gradually became more prevalent. All genotypes except V, XII and XIII were displaced by genotype XI starting on day 21 of the experiment, while on days 28 and 35 genotypes XII and XIII were not detected (Fig. 4b, Supplementary Fig. S1).

#### Recombination analysis of obtained PiCV strains

Recombination analysis of the PiCV sequences obtained in this study revealed 25 recombination events. Events 2, 4, 10, 11, 14, 21, 23 and 24 were detected before the start of joint housing of pigeons and occurred only in single sequences (except for event 24 occurring in 8 sequences originating from the same bird). Events 1, 5, 6, 12, 17, 19, 20 and 22 were detected both before and after the start of joint housing and occurred in numerous sequences belonging to genotypes I, II, VI, IX, XI, XII and XIII. After the birds were combined into one flock, events 3, 7, 8, 9, 13, 15, 16, 18 and 25 were detected. These recombination events usually occurred in single sequences, except for events 15 and 16, occurring in two and ten sequences, respectively. Recombinants that emerged after the start of joint housing were detected in birds originating from all flocks except IV and were only detected within the first 3 weeks of the experiment (13, 4 and 2 sequences recovered on days 7, 14 and 21, respectively). These recombinants belonged mainly to genotype XI (8 sequences), while the single recombinant sequences belonged to genotypes II, IV, VII and IX. Recombinants BV_3d_C4 and BV_1c_C1 were the only representatives of genotypes IV and IX, respectively (Table 1).

**Table 1:** A summary of the 25 recombination events detected using the RDP (R), GENECONV (G), BOOTSCAN (B), MAXCHI (M), CHIMAERA (C), SISCAN (S), and 3SEQ (T) methods implemented in the computer program RDP5.

Event	Number of sequences		Recombinant region		Recombinant sequence (s)/genotype	Minor parent sequence (s)/genotype	Major parent sequence (s)/genotype	Detection method	p-value
	Before commune housing	After commune housing	Begin	End					
1	31	31	1467	2038	BV_1c_C1/IX genotype I except: SI_1a_C4; SI_2a_C1-4; BI2b_C3	BV_3d_C4/IV	BV_3d_C9/XI	RGBMCST	3.69 × 10^–49^
2	1	0	1487	1958	SI_1a_C1/III	BI_2b_C6/II	SI_1a_C5/VI	RGMCST	4.79 × 10^–49^
3#	0	1	575	1487	BI_2b_C6/II	BI_2b_C8/II	SI_2a_C3/I; SI_1a_C4	GBMCT	1.69 × 10^–50^
4	1	0	378	1298	SI_1a_C5/VI	SI_1a_C1/I	SI_1a_C2/VI	GMCT	5.95 × 10^–33^
5	8	189	1894	614	genotype XI except: BII_2c_C4 and C8 BV_2a_C1 and C4 BV_2b_C9; BII_1d_C11	BII_2c_C8/ XI	BII_1d_C11/XI	RGBMCST	2.50 × 10^–30^
6	13	197	2021	179	Genotype XI except: BV_2b_C9; BII_1d_C11; BV_2a_C4; BII_2c_C4 genotype XIII (all)	Genotype VI except: BIII_3a_C1,C3,C5 and C10 SI_1a_C1/III	BII_1d_C11/XI; BV_2a_C4/XI; BV_2b_C9/XI	RGBMCST	7.55 × 10^–26^
7#	0	1	1441	2022	BV_3d_C4/IV	Genotype I: SI_2a_C1, 3, 4; SI_1a_C4; BI_2b_C3	BIV_1a_C5/V	RGMCST	5.87 × 10^–49^
8#	0	1	2011	99	BIII_2b_C2/XI	BIII_3a_C5/VI	BII_1d_C11/XI	RGBMCT	1.05 × 10^–21^
9#	0	1	1324	1820	BII_2c_C8/XI	SI_2a_C3/I	BII_1d_C11/XI	RGMST	1.30 × 10^–30^
10	1	0	1465	1622	BIV_3a_C10/X	BV_2b_C9/XI	BIV_3a_C8/X	GMCST	1.64 × 10^–22^
11	1	0	1959*	608	SI_1a_C1/III	SI_1a_C2/VI	SI_2a_C3/I BI_2b_C3/I	GBMCST	3.65 × 10^–32^
12	7	6	1195	2019	SI_1a_C1/III Genotype VI except: SI_1a_C5	Genotype VIII (all)	Genotype XI: BV_2b_C9; BV_2a_C1,C4; BII_2c_C4,C8; BII_1d_C11	RGBMCST	1.48 × 10^–15^
13#	0	1	73	574*	BII_2c_C4/XI	SI_1a_C5/VI	Genotype X (all)	RGMCST	1.56 × 10^–13^
14	1	0	2021	72*	SI_1a_C5/VI	Genotype XII (all)	SII_1c_C2/VIII	RGMCST	2.97 × 10^–12^
15#	0	2	110*	510	BIII_2b_C2/XI; BII_1d_C11/XI	BIV_3a_C6/I; BV_1a_C3/I	BII_2c_C4/XI	RGMCST	1.38 × 10^–11^
16	0	10	1203	1913	Genotype VII (all)	Genotype V (all)	Genotype I: SI_1a_C4; SI_2a_C1,C3,C4; BI_2b_C3	RGMCST	2.38 × 10^–14^
17	1	3	2019	149	BV_1c_C1/IX; BV_2b_C9/XI; BV_2a_C4/XI; BII_1d_C11/XI	Genotype I: SI_2a_C1, C3,C4; SI_1a_C4; BI_2b_C3; genotype II (all)	Genotype XII (all)	RGMCST	7.19 × 10^–9^
18#	0	1	1*	289	BV_1c_C1/IX	BIV_3a_C6/I	BII_2c_C4/XI	RGMCST	3.11 × 10^–10^
19	36	32	1*	137	genotype II (all); genotype VI (all)	genotype X (all)	BV_3d_C4/IV; BII_3a_C5/VI; genotype V (all)	RGMCST	5.65 × 10^–9^
20	4	3	1351	1897	Genotype I: SI_2a_C1,C3,C4; SI_1a_C4; BI_2b_C3; genotype II (all)	Genotype V (all)	Genotype XI: BV_2b_C9; BII_1d_C11; BII_2c_C4; BII_2b_C2; BV_2a_C1,C4; genotype XII except BII_3a_C8; SI_3a_C1/XIII; SI_3g_C3/XIII	RGMCST	2.62 × 10^–9^
21	2	0	2034*	73	SI_1a_C2/VI; BIII_3a_C5/VI	BV_3d_C4/IV; genotype V (all)	genotype XI: BII_2c_C4; BII_1d_C11; BV_2a_C4; BV_2b_C9	RGMCT	3.80 × 10^–7^
22	5	2	2022	72*	Genoptype XII (all)	BIV_1a_C3/V; BIV_2a_C4/V	BII_2c_C4/XI	RGMCT	1.94 × 10^–8^
23	1	0	192*	476*	SI_1a_C5/VI	BV_2a_C1/XI; BII_2c_C8/XI	SII_1c_C2/VIII	RGCST	7.84 × 10^–7^
24	8	0	1915	268	Genotype X (all)	Genotype VIII (all)	Genotype V (all)	RGBMCST	9.0,3 × 10^–7^
25#	0	1	510	765	BV_3c_C9/I	BV_2b_C9/XI; BII_1d_C11/XI	BIV_3a_C6/I	GBMCST	1.08 × 10^–7^

Only the detection methods with associated *p*-values < 0.05 are shown. For each recombination event, the method with the lowest *p*-value is underlined. ^#^Indicates recombination events that occurred after the start joint housing and are discussed in more detail in the article. *Indicates on indetermination of the actual breakpoint position.

**Figure 5:**
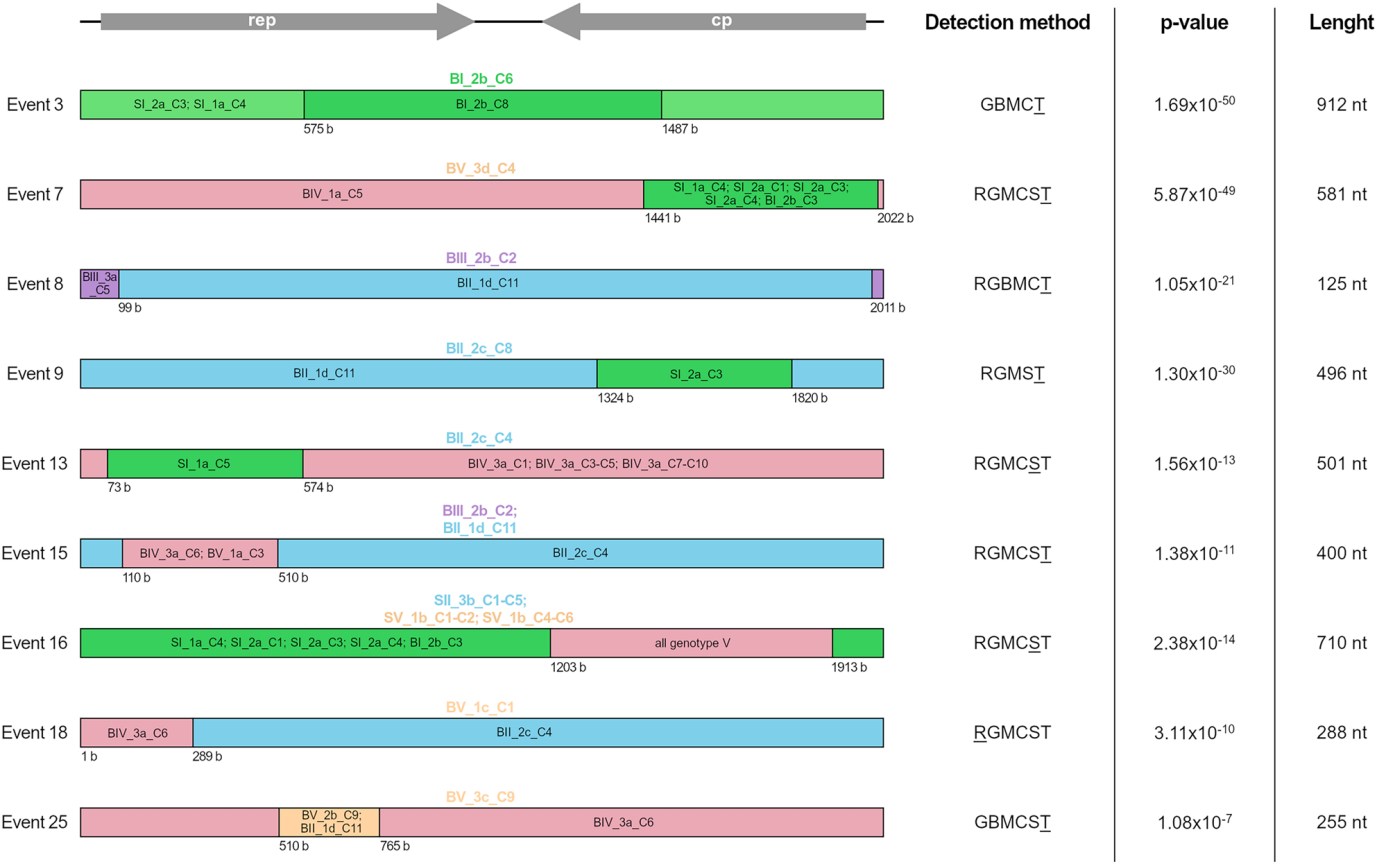
Schematic illustration of recombination events in complete PiCV genome sequences detected after the start of joint housing of experimental birds. The methods used to detect recombination events are RDP (R), GENCONV (G), BOOTSCAN (B), MAXCHI (M), CHIMERA (C), SISCAN (S) and 3SEQ (T). For each recombination event, the method with the lowest p-value is underlined. The color of the recombinant sequence names indicates the maternal loft of the pigeon from which the recombinant sequence has been obtained, whereas the color of the bars indicates the maternal lofts of the pigeons from which the parental sequences have been obtained: green—loft I, blue—loft II, purple—loft III, pink—loft IV and orange—loft V. The color coding was made according to Fig. 1. All sequence names are encoded with the following formula: B (blood)/S (swab), loft number (I–V), _pigeon number (1–3), sampling date (a–h), _clone number (C1–C10).

This study is focused on pigeon circovirus recombination occurring in young birds kept under OLR-like conditions. For this reason, recombination events detected after the start of joint housing are described in more detail. The recombinant regions of the genomes ranged in size from 125 to 912 nucleotides long, which represents approximately 6–45% of the size of the genome, and they were detected in 3 main genomic locations: (i) the intergenic region between the start codons of the *rep* and *cap* genes (event 8), (ii) the *rep* gene region (events 13, 15, 18 and 25) and (iii) the *cap* gene region (events 7, 9 and 16). The largest recombination region, which was event 3, was in the central part of the genome and partially overlapped *cap* and *rep* genes as well as involved the intergenic region between the stop codons of these genes (Fig. 5).

## Discussion

This experiment was primarily aimed at reconstructing the dynamics of the spread and evolution of PiCV, a virus which is highly prevalent in pigeons kept under OLR-like conditions, along with an attempt to determine the rate of formation of its recombinants. There were several reasons why PiCV was chosen as the research model. Firstly, this virus is widespread in the global population of pigeons including racing pigeons, fancy pigeons, meat pigeons and feral pigeons^7,20^. Secondly, it has a small genome which makes full genome sequencing relatively easy. Finally, genomic recombination which is a key mechanism for evolution is quite common in PiCV^8,11,17–20^. The main disadvantage which had to be taken into account when designing the experiment is that there have been no reports describing the isolation and propagation of PiCV in cultured cells^22^. For this reason, we decided to conduct an investigation on naturally infected pigeons, which accurately reflects the situation occurring in OLRs. The experiment was carried out in microscale due to the need to perform numerous analyses on a high number of samples. Conducting an experiment directly reflecting the number of birds in OLR, which usually ranges from hundreds to thousands, could result in difficult to evaluate sample numbers and very high costs. The experimental pigeons were PiCV-positive (most of the birds were viremic and all of them shed the virus with feces); however, they showed no symptoms of disease. Such a set-up of the experiment hinders the analysis of the obtained results significantly due to the obvious inter-individual differences in the values of the examined parameters. However, it is important to mention that pigeons in OLR are diagnosed and treated as a flock rather than individually. This was the reason for treating the experimental birds as one group during the analysis of the results. The values of all parameters were analyzed collectively and presented as a mean value of each parameter for all the examined birds on certain sampling dates. The results obtained on certain dates were then compared with each other. We are aware that performing such an experiment on a non-uniform experimental group of animals can lead to divergent values of examined parameters, which happened in this case, making the interpretation of the results difficult. Nevertheless, we have made every effort to ensure that the starting material used in the quantitative analyses is unified. Moreover, to make it easier to follow the dynamics of individual parameters tested in specific birds, we used color coding consistent for all figures.

Additionally, we selected the research methods with great care to present the research results as accurately as possible. Therefore, the ddPCR method was used to assess PiCV viremia and shedding. The development of modern laboratory techniques such as ddPCR allows direct quantification of genes without the need to plot a standard curve, which facilitates very precise quantitative analyses. According to Kojabad et al., ddPCR shows higher sensitivity and specificity than qPCR for the detection of viral nucleic acids^23^. Moreover, the tolerance to PCR inhibitors is enhanced in ddPCR, and this method is less dependent on the reaction efficiency than qPCR methods. What is also important, the initial target template concentration in qPCR is relatively higher than in ddPCR, which can result in false negative results.

The rate of virus replication and the time in which it is undetectable/below detectable levels in clinical samples largely depends on the cellular immunity of the organism^24^ Due to this fact, we have decided to investigate the potential correlation between the number of PiCV copies in peripheral blood and cloacal swabs and the level of expression of selected genes encoding cytokines associated with the antiviral response, such as interferon and interferon-stimulated genes (ISG)^25^. Moreover, due to the common occurrence of anti-PiCV antibodies in pigeon sera^12,26^, we decided to extend our research with serological tests to more fully illustrate the relationship between the course of natural infection with PiCV and the specific humoral response.

Genome sequence analysis of PiCV strains isolated during this study revealed the high genetic diversity characteristic of these viruses, allowing division into 13 genotypes. At the beginning of the experiment, the number of PiCV genotypes detected was the highest (9 genotypes detected on day 0 and 7 of the experiment). At other sampling dates, the number of genotypes detected oscillated from 2 to 5. From day 14 of the experiment, genotype XI became dominant. It is noteworthy that this genotype was not very prevalent at the beginning of the experiment, with a prevalence of approximately 10%. The number of complete PiCV genome sequences belonging to this genotype constituted 24% of the total sequences obtained on day 7 of the experiment and gradually increased to 90% at day 42. This genotype, originating from pigeon V_2, displaced all other genotypes throughout the experimental period. It could indicate the higher infectivity of strains belonging to this genotype, but this thesis cannot be fully confirmed. The main difficulty in the proper interpretation of this result is that, compared to the results of virus quantification with ddPCR (amplification of a small fragment of the genome), the recovery of complete PiCV genomes was relatively low and the complete genomes could not be obtained from numerous birds. However, in the case of pigeon III_2, we were able to recover complete genomes from each sampling date. The data obtained from this individual seems to reflect the general trend. Interestingly, on the first date of sampling, only sequences belonging to genotype I were obtained from this bird. On the second sampling date, 10 sequences from genotype I and 1 from genotype XI were recovered. On the other sampling dates, only sequences from genotype XI were derived from this bird, indicating that the other strains have been outcompeted by this genotype. This phenomenon was observed also in our previous pilot study^17^.

We detected several recombination events occurring in the PiCV genomes; however, the highest number of recombinants was present both before and after the start of joint housing. This indicates that most of the PiCV sequences detected before the start of the experiment were recombinants, which is not surprising in case of this virus^11^, but on the other hand may hinder recombination analysis. The main objective of this study was to assess the time course for the development of PiCV recombinants in young pigeons kept under conditions mimicking the OLR system. Therefore, we were mainly focused on recombination events occurring after the start of joint housing of experimental birds. Quantification of the virus with ddPCR revealed that the PiCV genome copy number in both blood and cloacal swab samples increased significantly during the first three weeks of the experiment. Nine new recombination events were detected during this period, which is consistent with our pilot study, in which 10 of 13 recombination events were detected after three weeks of joint housing of pigeons in a common loft^17^. The first novel recombinants emerged after only seven days of joint housing and the number of novel recombinants recovered on day 7 of the experiment was the highest among all samplings. The number of recombinant sequences harboring events qualified for analysis is not very high compared to the number of all recombinant sequences obtained in this study. However, we detected recombinant circoviruses in samples collected from six pigeons originating from all lofts except IV. Recombination analysis revealed that most of the “parental” viruses originated from samples collected on day 0 of the experiment (all recombinants harbored the partial genome of at least one virus recovered before the start of joint housing), and the remaining “parental” viruses originated from samples collected on days 7, 14 and even 21 of the experiment. The convergence of recombination prevalence with the viral loads in pigeon samples may suggest that the stress related to housing in new conditions promotes inter-individual cross-infections with various genetic variants of the same virus. This creates perfect conditions for development of recombinants, which was proven in this study. The novel recombinant sequences belonged to genotypes II, IV, VII, IX and XI. In the case of two of these sequences, the genotypes were represented with only one, recombinant strain. Interestingly, all novel recombinants were detected only on one sampling date, suggesting that these viruses were not able to replicate for an extended period of time. However, due to relatively low total number of complete PiCV genomes recovered from experimental birds, we are unable to fully explain this phenomenon.

The findings discussed above confirm the inter-individual cross-infection of certain viruses and multi-infection of the same host with various virus strains. This could be proof that the infection in a flock develops as a result of cross-infection with different strains and/or genotypes of the same virus in pigeons staying in a common room. Intensive replication and shedding of PiCV in the flock at the beginning of joint housing were probably related to the period of adaptation of the birds to the new conditions and the stress occurring during this period. Both viremia and virus shedding were the highest on day 14 of the experiment. After this date, the PiCV genome copy number found in cloacal swabs decreased by ca. 50% and was constant at this level within the two following weeks, before finally falling to almost zero at the end of the experiment. The similar pattern was noted in case of blood samples; however, the decrease of viral loads after day 14 was less sudden. Due to high standard deviation values, the differences in PiCV genome copy number were significant only in case of swab samples. The high value of standard deviation in all the investigated parameters at the beginning of the experiment possibly results from different origin of the pigeons, their various immune status and stage of infection. However, the clear decrease of the SD value in the last three weeks of the experiment definitely draws attention (Fig. 2b). This observation suggests uniform circovirus disappearance in all examined birds. Literature data allowing a thorough comparative analysis of our results is rather sparse. Nevertheless, the investigation conducted by Schmidt et al*.* (2008) shows some similarities in the achieved results. In the experiment carried out by this team, the PiCV genetic material was detected more often in cloacal swabs than in the blood on day 7 after inoculation. Moreover, the number of positive samples was the highest on day 14 of the experiment. Similar to our study, the number of PiCV-positive birds gradually decreased until the last day of the experiment. However, these authors did not conduct any quantitative analyses^22^. In both investigations most of the pigeons showed no clinical symptoms during the experimental period.

Also noteworthy is the fact that the PiCV genome copy number in the two pigeons which died during the experiment gradually increased until the day of their death, and at that time was the highest among the entire study group. The above is in accordance with our previous research, in which the PiCV genome copy number was the highest in the group of pigeons which were clinically infected with this virus^26^. Similar to our pilot study^17^, *E. coli* was found in the internal organs of dead pigeons, which is most likely related to the breaking of the immune system barrier in pigeons during the acute phase of circovirus infection. This explanation of such an occurrence was also suggested in the studies of other authors^27^. It should be emphasized here that numerous literature data indicate that clinical circovirus infections typically occur in pigeons under 1 year of age and are associated with lethargy, weight loss, diarrhoea, regurgitation and poor racing performance, what has been known as “young pigeon disease syndrome” (YPDS)^27,28^. However, it is currently believed that pigeon circovirus may not be the causative agent of this syndrome^29^, and that the main consequence of infection is immunosuppression^26^.

The innate immune response, mediated by interferon, is effective against numerous pathogens, including viruses. Once the pathogen is detected, the produced IFN molecules bind to receptors on the cell surface, initiating a signaling cascade resulting in transcriptional regulation of IFN-regulated genes^30^. The principal targets of IFN-γ are B and T lymphocytes, NK cells and macrophages^31,32^. One of the proteins activated by IFN and associated with antiviral response is the murine myxovirus resistance protein 1 (MX1), which was one of the first described inhibitors of viral entry. The broad inhibitory activity of MX1 is observed at an early post-entry step of the virus infection cycle, before the genome replication^30^. Moreover, the upregulation of this gene was observed in PiCV-infected pigeons treated with probiotic^35^. The comparative analysis of the PiCV genome quantification and the expression of genes related to anti-viral immunity shows a certain dependence—the IFN-γ related MX1 gene expression was inversely proportional to the PiCV genome copy number. However, this dependence was not confirmed statistically. This suggests that the viral replication and shedding inhibition could be strictly related to the cellular immune response of the host^33–35^. Further disappearance of the virus could be connected to development of the specific humoral response, because the highest level of anti-PiCV IgY was noted on day 28 of the experiment. The initial level of antibodies in experimental pigeons was similar to those noted during our previous research on field cases of PiCV infection^26^. The specific humoral response was relatively slow in the experimental birds. This may be related to its suppression, which was demonstrated during one of our previous investigations on reactivity of the immune system in pigeons with different PiCV immune status. During that research, a slower increase of the anti-PiCV recombinant capsid protein antibodies was detected in pigeons subclinically infected with PiCV than in uninfected birds^36^. At the end of the experiment, antibody levels decreased in comparison to day 28, which might be related to relatively low immunogenicity of circoviruses. Viremia and shedding decreased from day 21 of the experiment, so virus-induced stimulation was also lower, which in the case of weak immunogens, such as circoviruses, can lead to a decrease in antibody levels. A similar phenomenon was noted in the case of swine circovirus^37^.

The results have shown that the One Loft Race pigeon rearing system could play a significant role in spreading infectious agents such as circoviruses and contributing to PiCV evolution through creating the appropriate conditions for viral recombination. Therefore, it is worth considering, if pigeon racing, a popular gambling game, is sensible from both an animal welfare and epidemiological point of view.

## Materials and methods

### Ethics statement

The experiment was conducted with the use of domestic pigeons purchased from private breeding facilities. The research protocol was approved by the Local Ethics Committee on Animal Experimentation of the University of Warmia and Mazury in Olsztyn, Poland (resolution No 41/2019, issued 28.05.2019, valid through: 01.10.2023). All methods were carried out in accordance with relevant guidelines and regulations. Also, all methods were reported in accordance with ARRIVE guidelines. The researchers made every effort to minimize the suffering of birds.

### Birds

Fifteen pigeons of 4–5 weeks of age were bought from five private breeding facilities numbered I to V (three birds from each breeding facility) located in the Warmińsko-Mazurskie Voivodeship in Poland. PiCV infection has been detected in all of these facilities in the last five years. There were no connection including close localization, pigeon trade or visits of the owners between the lofts. The birds were qualified based on the general health status as well as parasitological, microbiological and molecular examination. All pigeons used for the experiment were clinically healthy and free of pathogenic fungi, bacteria and viruses; they were also positive for PiCV genetic material in the blood and/or cloacal swabs, except from one pigeon, originating from the loft II, which was negative in the PiCV screening.

### Experimental design

Prior to the start of the experiment, the pigeons were screened for the presence of PiCV genetic material in blood (viremia) and cloacal swabs (viral shedding) with the quantitative real-time PCR (qPCR) method described by Duchatel et al*.* (2009)^38^. The birds were then housed, fed and watered in one room for 6 weeks, enabling natural cross-infection with circulating PiCV strains mimicking the conditions of the OLR. The birds were kept in the Pavilion of Experimental Infections belonging to the Department of Poultry Diseases, Faculty of Veterinary Medicine, University of Warmia and Mazury in Olsztyn, Poland. This facility is equipped with a unique system of HEPA filters and an automatic control system maintaining a pressure cascade in corridors, boxes and sanitary locks that exclude the possibility of contamination of experimental rooms. During the stay of birds in the Pavilion, blood and cloacal swab samples were collected from each bird every seven days. The blood samples (1.5 ml) collected on days 0, 14, 28 and 42 of the experiment were divided into three parts. The first part was used for DNA extraction to enable the acquisition of complete PiCV genomes for sequencing and to quantify PiCV viral loads using digital droplet PCR (ddPCR). The second part was used for the isolation of mononuclear cells to evaluate the expression of IFN-γ and MX1 genes (qPCR), whereas the third part was used for obtaining serum to evaluate the antibody levels against PiCV (in-house enzyme-linked immunosorbent assay—ELISA). In turn, the blood samples collected on days 7, 21 and 35 of the experiment were divided into two parts for the aforementioned molecular analyses.

Cloacal swab samples were used for isolation of total DNA to quantify PiCV genome copies and thus assess viral shedding (using ddPCR) and for recovery of complete PiCV genomes for sequencing, in cases where genome sequencing from the blood samples was unsuccessful. The experimental design is illustrated in Fig. 1.

### DNA extraction

During the experiment, PiCV DNA was obtained from two types of samples: 20 µl of fresh whole blood collected in Vacutainer® EDTA tubes (Becton Dickinson, USA) and 350 μl of liquid medium in which the swabs were suspended (Copan Diagnostic, USA). Total DNA extraction was performed with an Extractme Genomic DNA kit (Blirt/ Qiagen, Germany) according to the manufacturer's guidelines. The concentration and purity of eluted DNA were measured with a NanoDrop 2000 spectrophotometer (Thermo Fisher Scientific, USA). The samples were then stored at − 20 °C until further analysis.

### qPCR screening for PiCV

#### Sample optimization

The eluted DNA samples have been standardized to nucleic acid concentration, so that each sample contained comparable amount of genetic material, which would increase the credibility of results acquired with quantification methods. DNA concentration of all samples has been measured with a NanoDrop 2000 Microvolume Spectrophotometer (Thermo Fisher Scientific, USA). The samples were then diluted with deionized water free of ribonucleases and deoxyribonucleases in such a way that the resulting concentrations were similar to the sample with the lowest concentration value of total DNA, which was 20 ng/µl in case of samples acquired from blood and 5 ng/µl in case of samples acquired from swabs. Subsequently, the DNA concentration was measured again to ensure that the samples were correctly diluted.

#### qPCR

The presence and quantity of PiCV genetic material in pigeons was determined by qPCR according to the method described previously^38^. The qPCR amplicons were 139 bp fragments of the replication-associated protein gene (Rep) and the mixture had the following composition: 10 μl of Power Sybr Green PCR Master Mix (Life Technologies, USA), 0.1 μl of primer YP09: 5 ꞌ -GGT ACC CGC ATA AGG TGC CCG T-3ꞌand 0.25 μl of primer YP10: 5 ꞌ -TTG ATC CGC CGG AAG AGC GCC T-3 ꞌ (both primers with a concentration of 10 μM), 7.65 μl of RNase-free water and 2 μl of eluted DNA. The reaction was carried out with the use of LightCycler® Real-Time PCR System thermocycler (Roche, Switzerland) under the following conditions: 95 °C for 10 min, followed by 40 cycles of three-stage amplification: 95 °C for 15 s, 64 °C for 30 s and 72 °C for 30 s. Samples were classified as PiCV-positive when Cq (cycle of quantity) values were equal to or lower than 35.

### Evaluation of PiCV viremia and shedding with ddPCR

The digital droplet PCR (ddPCR) was performed in accordance with the methodology previously implemented in the laboratory of Department of Poultry Diseases, Faculty of Veterinary Medicine, University of Warmia and Mazury in Olsztyn, Poland^26,36^. Prior to the analysis, all samples with an earlier evaluated Cq lower than 22 were diluted with distilled water according to the rule that a single tenfold dilution increases Cq by 3. This step was necessary to avoid oversaturation of PCR droplets, which could make proper quantification impossible.

The first step of ddPCR involved the preparation of 22 µl of reaction mixture, which consisted of 10 μl of QX200 ddPCR EvaGreen Supermix (Bio-Rad, USA), 0.22 μl of 10 μM forward and reverse primers the same as in qPCR, 2.2 μl of template DNA and 9.36 μl of nuclease-free water. The reaction mixture was transferred into individual wells of the disposable eight-channel DG8 cartridges (Bio-Rad, USA), which had already been loaded into a DG8 cartridge holder (Bio-Rad, USA) and the bottom wells were filled with 50 µl of QX200™ Droplet Generation Oil for EvaGreen (Bio-Rad, USA). Each prepared cartridge was then placed in a QX 200 droplet generator (Bio-Rad, USA). The obtained 40 μl droplet emulsions were further transferred into a semi-skirted PCR-clean 96-well plate. The plate was then heat-sealed with pierceable foil using a PX1 PCR plate sealer (Bio-Rad, USA), and PCR amplification was carried out in a C1000 Touch thermal cycler (Bio-Rad, USA). The ddPCR program consisted of initial 5 min step at 95 °C, followed by 40 cycles of 95 °C for 30 s, and 64 °C for 1 min with a ramp rate of 2 °C/s; the next cycle was performed at 4 °C for 5 min followed by 90 °C for 5 min. After the thermal cycling, the plate containing the droplets was chilled to room temperature and placed in a QX 200 droplet reader (Bio-Rad, USA) for viral genome copy number analysis. Each sample was analyzed in duplicate. In the case of diluted samples, the average result obtained from each repetition was multiplied by the dilution value. The results were expressed as mean PiCV genome copy number ± standard deviation (SD) per specified volume of the sample on each day of sampling.

### Amplification of complete PiCV genomes and genome recovery

Total extracted DNA of each PiCV-positive sample was enriched in circular DNA by rolling circle amplification (RCA) using (Cytiva, USA) as described previously^39–41^. The concatenated RCA DNA was used as a template to amplify PiCV genomes using “back-to-back” degenerate primers PiCV-NF 5'- TGA CTT CAA AAC GGA AGT CAT CGT CAT C -3' and PiCV-NR 5'- CGN GGC TGC TGA CCA ATC AGC -3' which targets the replication-associated protein gene. These primers can amplify the complete genomes of all PiCV variants^11,17^. The PCR amplicons were purified and cloned into pJET1.2 plasmid vector (ThermoFisher Scientific, USA), and the obtained plasmids were used for transformation of the competent *Escherichia coli* cells (XL1-Blue). The transformed bacteria were cultured on plates containing Luria–Bertani (LB) medium with ampicillin. The insertion of the PCR amplicon was confirmed by colony-PCR. Ten single colonies derived from blood samples and five single colonies derived from cloacal swabs, obtained from the transformation of each amplicon, were evaluated. Subsequently, all the positive colonies were inoculated into 5 ml of liquid LB medium with ampicillin and incubated overnight. Each colony was incubated separately. The DNA isolated from bacterial plasmids was extracted using the Fast-DNA spin plasmid purification kit (iNTRON technologies, South Korea) and Sanger sequenced by primer walking (Macrogen Inc., South Korea).

### Isolation of mononuclear cells

To isolate mononuclear cells 1 ml of the blood was mixed with the same volume of PBS and gently layered on 2.5 ml of Hitopaque-1077 (Sigma Aldrich, USA). After centrifugation (30 min, 400 × g, room temperature) the upper layer of the opaque of the interface containing mononuclear cells (m.c.) was carefully aspirated. The layer of m.c. obtained this way was rinsed twice and suspended in sterile PBS. Afterwards, the absolute lymphocyte count (ALC) was determined in each sample with a Vi-cell XR automatic cell viability analyzer (Beckman Coulter, USA).

### qPCR evaluation for IFN-γ and MX1 genes expression

#### RNA isolation

The number of mononuclear cells was standardized to 5 × 10^6^. Total RNA from these samples was obtained with a NucleoSpin RNA kit (Macherey–Nagel, Germany) following the manufacturer’s protocol. The concentration and purity of eluted RNA were measured with a NanoDrop 2000 spectrophotometer (Thermo Fisher Scientific, USA) and the samples were stored at − 80 °C until further analysis.

### qPCR

Extracted RNA was standardized to 0.5 μg per sample for reverse transcription which was carried out with a High-Capacity cDNA Reverse Transcription Kit (Life Technologies, USA) according to the manufacturer’s recommendations. The expression of genes encoding glyceraldehyde 3-phosphate dehydrogenase (GAPDH), interferon-ɣ (IFN-γ) and myxovirus resistance protein 1 (MX1) was determined by qPCR. The reaction mixture had the following composition: 10 μl of Power SYBR® Green PCR Master Mix (Life Technologies, USA), 0.6 μl (MX1) or 1.8 μl (GAPDH, IFN-ɣ) of each 10 μM primer, and 2 μl of cDNA. The final reaction volume was made up to 20 μl with RNase-free water. The primer sequences are presented in Table 2. The qPCR was performed using the following protocol: initial denaturation at 95 °C for 10 min, followed by 40 two-stage cycles: denaturation at 95 °C for 30 s, primer annealing and extension at 60 °C for 60 s. The relative expression of each gene was calculated using the 2 − ΔΔCt method^42^ normalized to efficiency corrections, expression levels of housekeeping gene (GAPDH) and reference group. Expression was analyzed with GenEx 6.1.0.757 software (MultiD, Sweden). To enable proper evaluation gene expression analyses, the mononuclear cells isolated from 4 PiCV-free pigeons were used as a reference control.

**Table 2:** List of primers used in the study.

Primer name	Sequence	Product size (bp)	Methods	References
GAPDH F	CCCTGAGCTCAATGGGAAGC	137	Relative qPCR	Dziewulska et al. 2018^53^
GAPDH R	TCAGCAGCAGCCTTCACTAC			
IFN-ɣ F	CTGACAAGTCAAAGCCGCAC	125		
IFN-ɣ R	AGTCATTCATCTGAAGCTTGGC			
Mx1 F	CAGCAGAGAGCTCCAGTATGC	135		Tsai et al. 2021^35^
Mx1 R	AGATCTGGGACATCTGGGGAC			
YP09 F	GGTACCCGCATAAGGTGCCCGT	139	Absolute qPCR, ddPCR	Duchatel et al. 2009^38^
YP10 R	TTGATCCGCCGGAAG AGCGCC			
PiCV_NF	TGA CTT CAA AAC GGA AGT CAT CGT CAT C	Complete PiCV genome	Sequencing	This study
PiCV_NR	CGN GGC TGC TGA CCA ATC AGC			

### In-house ELISA for detection of anti-PiCV IgG

The assay was conducted with an in-house ELISA test developed for the purpose of one of our previous studies^12^. The PiCV recombinant capsid protein (rCP) of the strain BV_2a isolated during this study was obtained as an external service in Genscript Biotech (The Netherlands) and used as a plate antigen. The test was performed on Nunc-Immuno Module plates (Thermo Fisher Scientific, USA) using an Antibody Pair Buffer Kit (Invitrogen, USA). Each well of the Nunc-Immuno Module plate was coated with PiCV rCP (concentration 20 µg/ml). Sera obtained from the blood of the examined pigeons were diluted 1:50 in the assay buffer, and deposited in each well in duplicate. Rabbit anti-pigeon IgG (antibodies-online GmbH, Germany) diluted 1:30,000 in the assay buffer were used as primary antibodies, and goat anti-rabbit antibodies with horseradish peroxidase (HRP) (BD Biosciences, USA) diluted 1:1,000 in the assay buffer were used as secondary antibodies. The rinsing after each step was performed with an ELx 405 automatic washer (Biotek, USA). Optical density was then measured with an ELx 800 spectrophotometer (Biotek, USA) at the wavelength of 450 nm. Data was expressed as mean OD_450_ ± standard deviation.

### Bioinformatic analyses

#### Evaluation of genetic diversity

The complete PiCV genomes were assembled using Geneious Prime v. 2023.2 software (Biomatters Ltd., New Zealand). The assembled sequences were aligned using MAFFT v7.113^43^. To show the genetic connections between the investigated strains, the alignment was used to generate neighbor-joining (NJ) tree of the complete genomes using the Tamura-Nei substitution model with 1,000 bootstrap replicates. All pairwise identity matrices were determined using SDT v1.2 software^44^. For the purpose of this study, pairwise identity of complete PiCV genome sequences < 97% was established as the threshold for genotype separation.

#### Recombination analysis

The recovered genomes were checked for the occurrence of recombination events using RDP5^45^ with all recombination detection methods available in this software: RDP^46^, GENECONV^47^, BOOTSCAN^48^, MAXCHI^49^, CHIMAERA^50^, SISCAN^51^, and 3SEQ^52^. Recombination signals associated with p-values < 0.05 which were detected by three or more methods and which had associated phylogenetic support for recombination were accepted as credible evidence of recombination events. Sequences in the analyzed dataset which most closely resembled the parental sequences of recombinants were defined as either ‘minor parents’ or ‘major parents’ based on the size of the genome fragments.

### Statistical analysis

The significance of differences between the sampling dates in all parameters tested was analyzed using repeated measures analysis of variance (ANOVA), followed by Tukey's test. Differences were considered significant at the confidence level of 95 (p < 0.05). All analyses were performed with Statistica 13.3 software (Statsoft, Poland).

### Supplementary Information

Supplementary Information 1.

Supplementary Information 2.

## Data Availability

Nucleotide sequences produced in the current study have been submitted to GenBank and are available under the following accession numbers: OR801794-OR802128 and OR999199-OR999278. Additional data is available upon request.
