# Supplementary figures and images for: The pigeon circovirus evolution, epidemiology and interaction with the host immune system under One Loft Race rearing conditions
Source: Sci Rep. 2024 Jun 15;14:13815. doi: 10.1038/s41598-024-64587-3 (PMC11178769; doi:10.1038/s41598-024-64587-3)

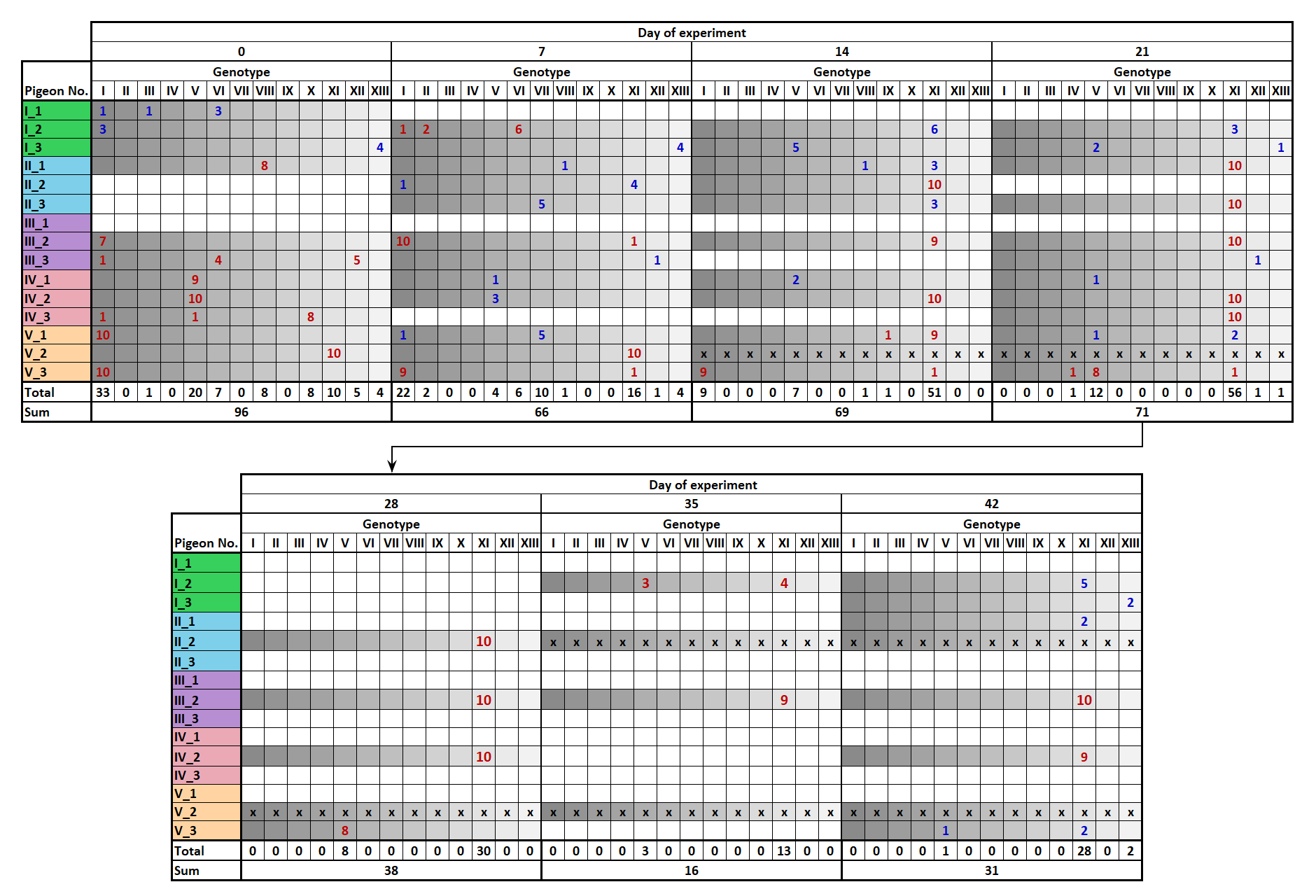

Supplement: Supplementary file 2 — Supplementary Information 2. [file 41598_2024_64587_MOESM2_ESM.png]
